# The youth sports club as a health-promoting setting: An integrative review of research

**DOI:** 10.1177/1403494812473204

**Published:** 2013-05

**Authors:** Susanna Geidne, Mikael Quennerstedt, Charli Eriksson

**Affiliations:** School of Health and Medical Sciences, Örebro University, Sweden

**Keywords:** Health promoting setting, organized youth sports, review, sports clubs

## Abstract

*Aims*: The aims of this review is to compile and identify key issues in international research about youth sports clubs as health-promoting settings, and then discuss the results of the review in terms of a framework for the youth sports club as a health-promoting setting. *Methods*: The framework guiding this review of research is the health-promoting settings approach introduced by the World Health Organization (WHO). The method used is the integrated review. Inclusion criteria were, first, that the studies concerned sports clubs for young people, not professional clubs; second, that it be a question of voluntary participation in some sort of ongoing organized athletics outside of the regular school curricula; third, that the studies consider issues about youth sports clubs in terms of health-promoting settings as described by WHO. The final sample for the review consists of 44 publications. *Results*: The review shows that youth sports clubs have plentiful opportunities to be or become health-promoting settings; however this is not something that happens automatically. To do so, the club needs to include an emphasis on certain important elements in its strategies and daily practices. The youth sports club needs to be a supportive and healthy environment with activities designed for and adapted to the specific age-group or stage of development of the youth. ***Conclusions*: To become a health-promoting setting, a youth sports club needs to take a comprehensive approach to its activities, aims, and purposes.**

## Introduction

Sport is one of the most popular leisure-time activities among young people, and is often organized in the form of sports clubs or extracurricular athletics at schools [1,2]. Because of the increasing number of children and young people participating in organized sport all over the world, sports clubs and young athletes have become an important target for societal interventions and policies of different kinds, not least in relation to health [2–4]. The topic has also attracted attention within the research community, and some research has shown an interest in youth sports clubs as sites for health promotion, because of the societal potential of youth sports clubs to work more health promoting and the large number of adolescents they engage. But what does this body of research reveal?

Recent reviews within the wider research area have focused on benefits and outcomes of physical education and school sports [3,5], on policy interventions in sports organizations [4], or on examining the physiological health benefits of participating in sports [6–8]. However, health, as well as the relation between sports and health, can be conceived of in many different ways [9–12].

The framework guiding this review of research is the health-promoting settings-approach introduced in the Ottawa Charter on health promotion, which states that ‘health is created and lived by people within the settings of their everyday life, where they learn, work, play and love’ [13, p. 3]. The concept of healthy settings has been developed during the past 20 years and is now a key element of several public health strategies [14]. It is used in a variety of areas, for example, healthy cities [15], healthy schools [16] and healthy workplaces [17]. However, the settings-based approach related to sports clubs is not an explicitly well-studied area, even though sports organizations have the potential to promote and create health- promoting environments [4,18,19]. There are also other models with an ecological approach that have frameworks in different contexts, such as Bronfenbrenner [20] in psychology, Dooris [14] with a settings-based approach and Rütten and Gelius [21] in health promotion with influences from Giddens, among others. However, the framework in this review is a health-promotion model with a settings-based approach that takes influences from other disciplines, which can strengthen the framework for youth sports club as a health-promoting setting.

The ambition of this review is consequently to contribute to further knowledge development in this evolving area of research, and the aim of this integrative review is twofold: first to compile and identify key issues in international research about youth sports clubs as health-promoting settings; and second, to discuss the results of the integrative review in terms of a framework for the youth sports club as a health-promoting setting.

## Background

### The youth sports club

Sport is a global phenomenon. Possibilities to participate in organized sports have radically increased, and politicians as well as researchers advocate the benefits of young people’s participation in sports [1,2,22].

One obvious, but also contested, outcome of organized youth sport is increased physical activity. It is widely known that physical activity has health benefits such as reducing the risk of cardiovascular diseases and different kinds of cancer as well as reducing obesity [23]. Other positive outcomes of physical education and sport for children’s development can be, as Bailey [5] suggests, within the five domains; physical, lifestyle, affective, social and cognitive. It is also argued that participation in organized youth sports in childhood can predict physical activity in early adulthood. For example, Kjønniksen and colleagues [24] conclude that if young people are members of sports clubs from an early age, they are more likely to be physically active in early adulthood, especially if they also are members during adolescence. Engström [25], however, in a 38-year follow-up study, found different results. He concludes that neither membership nor amount of time spent on sports at the age of 15 has any significant association with exercise habits in middle age. Engström’s study instead indicates that a broad and varied experience of sports during childhood and adolescence has an impact on exercise habits later on in life. Engström shows that the middle-aged person’s exercise habits are instead closely connected to their cultural capital at the age of 15, as well as their social position and educational capital later in life [25].

However, organized youth sports are not just physical activity. Research also suggests that it can be an arena for developing social skills like cooperation, responsibility, empathy and self-control, as well as promote good citizenship, social success, positive peer relations, leadership skills, and a sense of initiative [3,26,27]. Highly structured leisure activities, such as organized youth sports, are also linked to low levels of antisocial behaviour, and the presence of supportive adults, non-deviant age-mates, and clear activity goals in structured activities could improve individual, social and physical competencies and promote positive adjustment [28]. Engström [29] further argues that membership in a sports club does not only improve sporting skills, but also social training – the learning of rules, norms, values, and lifestyle; it can furthermore promote a positive attitude toward one’s own body. Rutten and colleagues [30] describe organized youth sports as a positive socialization context.

Participating in organized youth sports provides opportunities for positive outcomes, but they do not necessarily come automatically [31]. A prerequisite for youth to benefit from organized sport is that they participate, and consequently that organized sports are accessible for all youth [32]. In this matter, research shows that the idea of equal opportunities to participate is highly problematic and that participation is dependent on both gender and socio- economic factors [33–35].

There are of course also negative outcomes of sports, for example, sports-related injuries, eating disorders, or pressure to win [26]. Omli and LaVoi [36] also show that young people could be negatively affected by the behaviour of angry parents in sports environments. Youth sports today are more than ever characterized by early specialization, being overly serious and organized, competition at earlier ages, and earlier selection [26]. It is, for example, harder than before to be successful in more than one sport, and the variation between physically active and inactive youth is more distinct. Timpka and colleagues [37] further ask if competitive sport is compatible with the best interest of the child according to the UN Convention on the Rights of the Child, and the authors discuss the concepts of sports trafficking, child labour and doping as negative outcomes of youth sports. Also more negative outcomes concerning alcohol and other drugs have been discussed by for example Lisha and Sussman [38] and Wichström and Wichström [39].

There is consequently a disparate picture of the benefits and the drawbacks of young people’s participation in organized sports, not least in terms of health. In this integrative review of youth sports clubs as health promoting settings, the youth sports club needs to be clarified in two ways. The review is dealing with organized sports clubs primarily for children and adolescents, not sports clubs for professional athletes, which can be seen more as commercial companies [40]. Then there is the concept of a club for sports. Youth sports are organized differently in different parts of the world, and the word “club” has different meanings [2,22]. This review is restricted to organized youth sports outside of regular school curricula. This means that the sport activity can be organized both through community and non-governmental organizations, but also by schools as an optional after-school activity. The important demarcation in the review is that it must concern young people’s voluntary participation in some sort of ongoing organized athletic activity.

### The settings-based approach to health

As illustrated above, health and the relationship between sports and health can be approached in diverse ways. In this review, we have adopted the World Health Organization’s (WHO) perspective on health promotion and the settings approach. Health promotion, which is the concept underlying the settings approach in the influential Ottawa Charter, is defined as “the process of enabling people to increase control over, and to improve their health” [13, p. 1].

From this definition, the concept of health promoting settings is based on the idea that changes in people’s health-related behaviours are best achieved through a focus on “the settings of their everyday life; where they learn, work, play and love” [13, p. 3]. The approach is consequently not primarily directed towards actions focussing on individual skills and behaviours, but instead towards actions directed at changing environmental conditions and organizational cultures [19]. In this way it is not just physical activity levels or individual skills that can make a sports club a health-promoting setting, but instead other factors such as young people’s social and mental well-being, as well as environmental factors like physical environment, policies, pedagogies, and social relations.

Kokko and colleagues [19] state that a settings-based approach includes two important dimensions: “a setting constitutes the context within which and through which health appears; and (ii) a setting offers an effective way to study and understand the determinants of health and to attain and influence individuals and communities” [19, p. 220].

In this vein, the Ottawa Charter [13] identifies five strategic imperatives for health promotion: 1) *Build healthy public policy* – which is about legislation, organizational change and policies that foster equity and ways to make a healthy choice the easy choice; 2) *Create supportive environments* – which describes ways in which society and parts of society organize work and leisure to be safe, stimulating, satisfying, and enjoyable; 3) *Strengthen community action –* which involves setting priorities, making decisions, and using strategies that empower a certain community through self-help and social support; 4) *Develop personal skills –* which is about supporting personal and social development through information, education, and the development of life skills; and finally 5) *Reorient health services –* which includes transforming the health care system in the direction of health development and the cultural needs of the population. Although the Ottawa Charter has been a cornerstone in shaping public health practice, the world has changed in many ways since it was written.

The Bangkok Charter [41] emphasizes the globalized world and adds, for example, that settings must form partnerships and build alliances with all sectors including nongovernmental organizations.

In this integrative review, health is conceptualized in line with WHO’s approach to health promotion and health-promotion settings, and an attempt is made to go beyond the idea of sport as promoting health through physical activity, towards an idea of the sports club as a health-promoting setting. This means that the review includes research that explores and discusses youth sports clubs in terms of an explicit and/or implicit focus on creating and providing prerequisites and environments for health promotion [13].

## Methods

### The integrative review

The integrated review, the method used in this paper, is the broadest of the review methods; it allows for both quantitative and qualitative studies and both theoretical and empirical literature. Its purpose is to review methods, theories, and/or empirical studies concerning a particular topic [42]. It can also be defined as a method that summarizes past research and draws conclusions from it [43]. Results of research reviews can be presented as summaries, analysis or synthesis. Summing up, one can, following Whittemore [42], say that a summary describes findings by identifying categories, an analysis adds critique of methods or outcomes, and a synthesis also includes creating a new model or organizing a framework for the topic of interest. Compared to the quantitative meta-analysis, a synthesis does not aim to aggregate data to produce statistical cause-and-effect relationships; instead, the synthesis employs new ways of conceptualizing the research field [44].

This particular review consists of both quantitative and qualitative studies and also both empirical and theoretical literature. The aim of the review is not to detect a statistical relationship between, for example, sports clubs and health. Instead, the first aim is to compile and identify key issues in international research about youth sports clubs as health-promoting settings. The second aim is to synthesize the results of the integrative review in terms of a framework for the youth sports club as a health- promoting setting.

### Material

A search of English-language publications was made in October 2010 in the full-text databases ABI/ inform, ACS – American Chemical Society, Blackwell Synergy, Cambridge Journals Online, DOAJ, EBSCO/ Academic Search Elite, Emerald, JSTOR, MUSE, Oxford University Press, Journals Online, Sage, ScienceDirect (incl. backfiles psychology), Springer and Wiley, for the years 2000 to 2010. This period of time was chosen to obtain a reasonable number of recent studies.

The primary keywords used were “youth sport” and “youth sports”, 131 and 243 studies respectively, and both were combined with “health”, 10 and 23 studies respectively. This quite wide concept was used because of the differences in how youth sports are organized throughout the world, including sports for both children and adolescents. The inclusion criteria for the review were, first, that the studies concerned sports clubs for young people (under 18 years), not sport clubs for professional athletes; second, that it should be a question of voluntary participation of some sort (both recreational and competitive, single- or/and multi-gender) of ongoing organized athletics outside of the regular school curricula (this also excludes research on short-term youth sports programmes, which can be seen more as temporary interventions); and third, that the studies consider issues about youth sports clubs in terms of health-promoting settings as described by WHO. Hence, studies with, for example, an exclusively individual perspective were excluded, as were studies concerning solely physical activity, for example, studies of no relevance for settings as described above. The fact that youth sports are organized quite differently around the world made it difficult to separate non-governmental from community-organized sports in the review. However, in relation to the purpose of the review concerning the settings-based approach, the variation of how youth sports is organized with for example paid personnel or volunteers should not have affected the results. Search hits without references from non-scientific publications and brief conference contributions were also excluded. This initial search resulted in a total number of 30 studies.

Furthermore, the reference list of included studies was examined and a search in Google Scholar was tested to identify additional articles not included in the initial search. This so-called snowballing method added 22 studies to the review, making 52 studies altogether. The snowballing method together with the search between 2000 and 2010 makes it reasonable to assume that we have included recent articles as well as important well-cited publications from earlier periods in the review. Eight studies were subsequently excluded in a further reading because they did not fulfil the inclusion criteria of relating to health promoting settings. The final sample for the review consists of 44 publications, one from a book, two from Swedish reports, and the rest from peer-reviewed journals. In Table I, all included publications are presented under the strategic headings of the Ottawa charter with a brief content description and country of origin, and also their relevance for health promoting youth sports clubs.

**Table I.** The review publications presented under the strategic headings of the Ottawa Charter with a brief content description, country of origin and relevance for health promoting youth sports clubs. Single publications are occasionally situated under more than one heading.

Ottawa categorisation	Authors	Brief content Country of origin	Relevance for HPYSC
**Build healthy public policy**	Casey et al. (2009)	Health-promotion capacity of sport organizations.	Health-promoting setting approach in sport organizations.
		Australia	
	Crisp and Swerissen (2003)	Creating health promoting sport environments.	Health-promoting setting approach in sport organizations.
		Australia	
	De Knop et al. (2004)	Quality management in sports clubs.	A tool for internal quality-checks in sports clubs.
		Belgium	
	De Knop and De Martelaer (2001)	Quantity and quality of youth sports.	Quality of youth sport.
		Belgium/The Netherlands	
	Dobbinson et al. (2006)	Health-promotion policies in sports clubs.	Health-promoting setting approach in sports organizations.
	Australia		
	Engström (2008a) (National report)	The Swedish initiative Handslaget.	Grants to sports clubs for e.g. policy work and community cooperation.
	Sweden		
	Le Menestrel and Perkins (2007)	Sports-based youth development.	Positive youth development.
	USA		
	Priest et al. (2008) (Review)	Policy intervention in sports organizations.	Policy intervention in sports organizations.
**Create supportive environment**	Côte and Hay (2002) (Book)	Children’s involvement in sport.	Youth’s socialization into sport.
	Daniels (2007)	Cooperation versus competition	Cooperation for positive youth development
		USA	
	De Knop et al. (1999)	Design of organized youth sports.	Design of youth sport – sport schools.
		Belgium	
	De Knop and De Martelaer (2001)	Quantity and quality of youth sports.	Quality of youth sport.
		Belgium/The Netherlands	
	Eime et al. (2008)	Sports clubs as health and welcoming environments.	Health-promoting setting approach in sports organizations.
		Australia	
	Engström (2008b)	A 38-year follow-up study about sports participation.	Sporting breadth during earlier years results in later good exercise habits.
		Sweden	
	Engström (1996) (Book)	Swedish trends in youth sport.	Sports schools.
		Sweden	
	Fraser-Thomas and Côte (2006)	Benefits of youth sport participation.	Design of youth sport.
		Canada	
	Fraser-Thomas et al. (2008b)	Adolescent sports dropout. Canada	Design of youth sports – diversity of sports.
	Fraser-Thomas et al. (2005)	Positive youth development through sport.	Design of youth sport. Coaches and parents importance.
		Canada	
	Fraser-Thomas et al. (2005)	Positive youth development through sport.	Design of youth sport. Coaches and parents importance.
		Canada	
	Griffin (2008)	Myths in sports education	Multisport participation
		USA	
	Hill and Green (2008)	Design of youth sport Australia	Modify children’s sports to fit children.
	Le Menestrel and Perkins (2007)	Sports-based youth development.	Positive youth development.
		USA	
	MacPhail et al. (2003)	Youth’s socialization into sport. UK	Multi-sports club
	Scheerder et al. (2006)	Sports participation among females.	Diverse sport pattern.
		Belgium	
	Wagnsson (2009) (Dissertation)	Organized sports as socialization arena (Swedish).	Design of sport.
		Sweden	
	Wiersma (2001)	Sources of enjoyment in sports.	Sources of enjoyment in sports.
		USA	
	Wiersma (2000)	Risks and benefits of youth sports specialization.	Sources of enjoyment in sports.
		USA	Coaches’ and parents’ importance.
**Strengthen community action**	De Knop and De Martelaer (2001)	Quantity and quality of youth sports.	Quality of youth sport.
		Belgium/The Netherlands	
	De Knop et al. (1996) (Book)	Worldwide trends in youth sport.	Design of youth sport.
	Engström (2008a) (National report)	The Swedish initiative Handslaget.	Grants to sports clubs for e.g. policy work and community cooperation.
		Sweden	
	Flintoff (2008)	School sports partnerships. UK	Cooperation between schools and sports clubs.
	Flintoff (2003)	School Sports co-ordinator programme.	Cooperation between schools and sports clubs.
		UK	
	Kirk (2005)	Youth sports and lifelong participation.	Design of youth sport.
		UK	
	Kokko et al. (2006)	Health-promoting sports clubs. Finland	Health-promoting setting approach in sports organizations.
	Persson (2008)	Social capital in sport. Denmark	Sports organizations social policy agenda.
**Develop personal skills**	Bloom et al. (2008)	Coaches’ role in team building. Canada	The coaches’ competence needs.
	Coatsworth and Conroy (2006)	Coach training. USA	The coaches’ competence needs.
	De Knop et al. (1999)	Design of organized youth sports.	Design of youth sport – sport schools.
		Belgium	
	De Knop and De Martelaer (2001)	Quantity and quality of youth sports.	Quality of youth sport.
		Belgium/The Netherlands	
	Engström (1996) (Book)	Swedish trends in youth sport.	Sports schools.
	Fraser-Thomas and Côte (2006)	Benefits of youth sport participation.	Design of youth sport.
		Canada	
	Fraser-Thomas et al. (2008a)	Dropout in adolescent competitive sport.	Coaches and parents importance.
		Canada	
	Fraser-Thomas et al. (2005)	Positive youth development through sport.	Design of youth sport. Coaches and parents importance.
		Canada	
	Fry and Gano-Overway (2010)	Caring climate in youth sport. USA	Coaches’ importance.
	Gould et al. (2007)	Coaching life skills through football.	Coaches’ importance.
		USA	
	Gould and Carson (2008)	Life skills through sport.	Coaches’ education.
	Guivernau and Duda (2002)	Moral atmosphere in sport	Coaches and parents importance.
	Kokko et al. (2006)	Health-promoting sports clubs. Finland	Health-promoting setting approach in sports organizations.
	LaVoi et al. (2008)	A parent education program.	Parents’ education.
		USA	
	Lundåsen (2005) (National report)	Voluntary contributions and health (Swedish).	Coaches’ health.
		Sweden	
	Omli and LaVoi (2009)	Anger in youth sport. USA	Angry parents.
	Rutten et al. (2007)	Organized sports and antisocial and prosocial behaviour.	Relations with coaches.
		The Netherlands	
	Wiersma (2000)	Risks and benefits of youth sports specialization.	Sources of enjoyment in sports.
		USA	Coaches’ and parents’ importance.
**Reorient health services**	Frisch et al. (2009) (review)	Injuries and prevention initiatives in youth sports.	Injury prevention.
		Luxembourg/Belgium	
	Timpka (2008)	Sports safety.	Sports safety.
		Sweden and others.	
	Weaver et al. (2002)	Preventing sports injuries. USA	Holistically-structured prevention approach.

### Analysis

**Figure 1.**
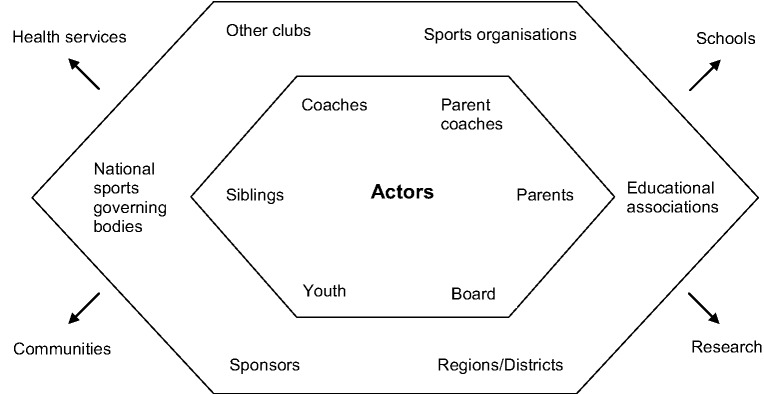
A multi-stakeholder approach illustrating the different actors of a health-promoting youth sports clubs including cooperation with other settings (influenced by Dooris, 2004 [14]). (Inner circle = Internal, Outer circle = External, and Outside = Other settings)

**Figure 2.**
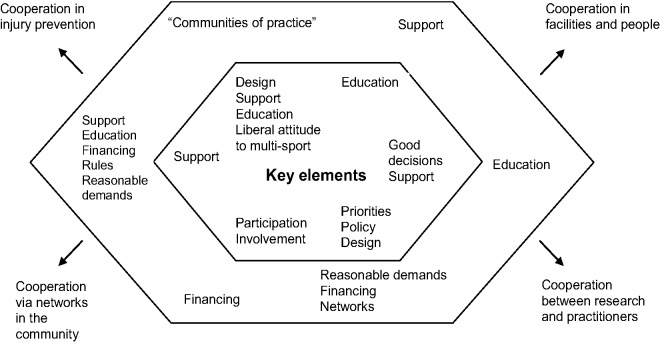
Examples of the key issues from the review illustrated in what the different actors could work with to build a health-promoting sports club. The different internal and external actors as well as other settings emanate from Figure 1. Hence, the figure could with advantage be read in connection with the different actors in the multi-stakeholder approach illustrated in Figure 1.

Our analytical steps in the study were: (i) Abstracts being from non-scientific publications and brief conference contributions were excluded. (ii) Each abstract using the primary keywords were read. (iii) The ones who clearly did not follow the inclusion criteria were excluded; the other studies were read through as a whole. (iv) A snowballing method was used to add further studies; this method also included older studies, which were referred to in the newer ones, to get the important contributions which were not in the review’s period of time. (v) The studies, which followed the first two inclusion criteria, were read through and the ones also followed the third inclusion criteria were included in the study. (vi) The components identified to be meaningful for the youth sports club as a health-promoting setting were categorized under the strategic headings of the Ottawa Charter. (vii) The components under each category were further compared and some were rearranged. (viii) The key components to the frameworks (Figures 1 and 2) were identified from the results of the review, based on Dooris’s [14] multi-stakeholder approach adapted to the setting of health promoting youth sports clubs.

In order to secure the trustworthiness of the study, the authors have continually discussed the different components, the categorizations as well as the results as a whole.

## Results

### Key issues about youth sports clubs

The different key issues about youth sports clubs as health-promoting settings that were found in international research are presented under the five strategic areas of health promoting settings: (i) Building healthy public policy, (ii) Creating supportive environments, (iii) Strengthening community actions, (iv) Developing personal skills, and (v) Reorienting health services.

#### Building healthy public policy

Under the area building healthy public policy, youth sports clubs can encompass changes in legislation at a central level, organizational change at national, regional, or local levels, or the writing of policies at different levels. What the changes in legislation, organization, or policies have in common is that they all put health on the agenda.

Priest and colleagues [4] conclude that policy development in sports settings is an understudied aspect of health promotion; however, policy development regarding sports clubs has been found to provide health benefits to the larger community [45]. In creating a health-promoting setting, it is important to focus on changing the whole organization to meet the new challenges, and, according to Casey and colleagues [46], it seems important that clubs be solidly organized. This is sometimes challenging to accomplish, however, with many conflicting wills of engaged parents and unpaid volunteer coaches. To create policies for the sports clubs, different types of actions can be identified as ways to improve the organization. De Knop and colleagues [47] speculate that in order to survive, traditional sports clubs are forced to be more professional. His research team has designed a web-based internal quality-check for sports clubs, called IKSport (in Dutch). The results from using this instrument in the Netherlands reveal that only half of the studied sports clubs had considered writing down their objectives, and only half of the sports clubs involved their young members in the club leadership. More than a third of the clubs had a shortage of board members, however, most of the sports clubs had a functioning system for internal communications.

Crisp and Swerissen [48] further argue that in order to get widespread support for the local policy in the club it is important to work with the policies. It also seems important that the youth sports club have a youth-oriented approach in terms of the setting being youth-centred [31,49]. A youth-centred approach includes, for example, involving young people and giving them responsibility, starting youth committees, letting the young participate at their own level, and giving them opportunities to make a difference.

Research from Sweden [50] reveals that there are several examples of specialized sports federations or clubs consciously working with their internal policies. In some cases, special government grants, administered by the Swedish Sports Federation in a special national programme called “Idrottslyftet” (A Lift for Sports, formerly “Handslaget”, a Handshake with Sport) have made this effort possible.

#### Creating supportive environments

Creating supportive environments involves organizing youth sports clubs to be safe, stimulating, satisfying and enjoyable, as well as not separating health from the other goals of the club. It also involves the quality of youth sports, how the practices of youth sports are designed, and how young people are socialized within and into sports.

De Knop and De Martelaer [49] conclude that health promotion in youth sports is not just about increasing the amount of participation in sports, but also improving its quality. Quality is discussed in terms of schools and organized sports cooperating, young people being involved in deliberate choices, sports being adapted to the youth, and club staff being trained in a sound way. The design of organized youth sports could, according to De Knop and De Martelaer [49], be a major factor in whether young people obtain positive or negative outcomes from it. Fraser-Thomas and colleagues [27] argue that programme design and the influence of parents/coaches are the two most important factors for positive and/or negative outcomes of youth sports, and Le Menestrel and Perkins [31] and Daniels [51] state that a positive youth sports climate depends on parents, coaches, officials, and youth working together with the community. In this vein, Wagnsson [52], in a Swedish study, concludes that the socialization effects of sports on young people’s pro-social development are quite small, but that organized sports nevertheless have the potential to serve as a positive socialization arena. Engström [29] on the other hand states that youth sport is one of our most important environments for the socialization of young people.

Building on Côte and Hay [53], MacPhail and colleagues [54] argue that the socialization of young people into sport follows a certain pattern; however this theory could also be seen as a discussion of the different pathways that individuals can take for athletic development. The theory comprises several phases of youth participation in sport. These are sampling (6–12 years), specializing (13–15 years), recreation and investing (16+ years). Following these phases and not rushing ahead too soon can, according to Côte and Hay [53], be significant for whether young people keep on doing sports or drop out. There is also research that states that sport environments that include play and involvement in a variety of activities, instead of specialization and deliberate practice, are in many ways better in terms of continued motivation for participation, physical competence and enjoyment [26]. Wiersma [55] also concludes that the potential risks outweigh the potential benefits when it comes to early specialization for young people, and Engström [25] similarly concludes that experiencing a broad variety of sports when young seems to have an impact on middle-age exercise habits.

MacPhail and colleagues [54] emphasize along these lines that fun and enjoyment can have different meanings and styles in different ages and phases. The fun of participating in sports consists, for younger people, of social elements and play; while for those who have started to specialize in a sport, it is more about the excitement of competing and performing well [54]. Wiersma [56] further argues that the two most important sources of enjoyment in youth sports are personal performance-mastery and competitive challenge, but social influences, such as affiliation with peers and positive adult involvement, are also mentioned in the study.

The review further reveals that there are opportunities and possibilities to make the organization and environment more supportive and health-promoting. Eime and colleagues [18] describe an Australian programme that is trying to establish healthy and welcoming club environments (HWE) by implementing practices and policies as a means to promote participation in sports. Results show that creating HWE clubs will lead to increased participation in sports. It also seems important that the young people in the sports clubs be involved in the process of creating supportive environments and producing policies because, on the one hand, they have to be part of the new ideas to support them and, on the other hand, the new ideas will thereby be their own [49]. De Knop and De Martelaer [49] also suggest that youth sports clubs should adapt the activities to young people in all possible ways. Rules in sports are primarily made to fit adults, not only when it comes to the size of the goals and length of the matches, but in time actually spent playing as well as the character of the training sessions, which sometimes are to monotonous [49]. This, according to Hill and Green [57], limits young people’s opportunities to obtain benefits from sports.

Some organizational ideas have been proposed in different countries that deal with the design of youth sports, for example, Engström’s [29] Sport Schools, De Knop and colleagues’ [1] Sport Academies, and Macphail and colleagues’ [54] Multi-Sports Club. The idea of “sport schools’ emerged in Sweden in the 1980s as a reaction to the debate about early specialization and overly serious youth sports. These organizations provide a place where children can be offered many different sports in a playful way [29]. In the Belgian region of Flanders, there are sport academies that are similar to the Swedish sport schools. The young people are not required to be members, but can try different sports in a welcoming environment [58]. MacPhail and colleagues [54] refer to “multi-sports clubs” which provide a variety of activities for younger children without requiring them to be members. Fraser-Thomas and colleagues [59] further argue that children should be encouraged to participate in a variety of playful sports, that coaches must emphasize reasonable practice schedules to allow children to be involved in other activities, that a fun and motivating climate should be created, and that specialized training should be delayed. Additionally, Griffin [60] concludes that participating in multiple sports is beneficial to young athletes, improves their fundamental motor skills and helps prevent burnout and overuse injuries. Scheerder and colleagues [61] state on this topic that a highly diverse sports pattern during late adolescence (at least for females) gives better opportunities for active participation as adults (see also Engström [25]).

#### Strengthening community actions

A youth sports club is always part of a community, through its participants, premises, funding, and so on. Under the area strengthening community action, it is important for youth sports clubs to cooperate with schools or other actors, but also for other actors in the community to see youth sports clubs as possible partners to develop and strengthen the community’s health- promotion work.

One way to achieve these goals, and at the same time increase the participation in and the quality of organized youth sports, is, according to De Knop and De Martelaer [49], to develop the cooperation among different youth sports clubs and between youth sports clubs and the schools. At present it is more a question of rivalry between sports clubs than actual cooperation [1].

An example of beneficial cooperation between schools and sports clubs, reported by Flintoff [62,63], is the UK School Sport Partnership Programme which encourages schools and community sports providers to work in partnership. This programme has resulted in more opportunities for sports activities, both in and outside the schools, as well as more qualified teachers involved in sports outside the schools [62,63]. The Swedish “handshake” programme also presents several examples of cooperation between schools and sports clubs, revealing that the efforts were appreciated but that the form of the collaboration needs to be further looked into. One result is that sports seem to have moved into the school without any particular adaptation, and mostly reaching pupils already active in sports [50]. On this subject, Kirk [64] compellingly argues that two major challenges for the cooperation between schools and local multi-sports clubs are increased sharing of facilities and cooperation between PE-teachers and coaches, especially in the initial sampling years.

In some parts of the world, such as the Nordic countries, youth sports clubs receive financial support from the state and the municipalities. This involves both obligations and opportunities for the sports clubs, in terms of what can be expected from them and how [19]. Persson [40] makes a case for there being greater pressure on publicly-funded sports associations to deliver in terms of the social policy agenda, but also that improving the social responsibility of sports clubs could lead to their obtaining greater social capital, new memberships and assurances of future financial and social support.

#### Developing personal skills

A youth sports club is a setting where young people as well as parents and coaches can develop personal skills. In sports contexts, these skills are often described in terms of motor skills and their improvement. In a health-promoting settings approach, a wider picture is considered that includes personal and social development through information and education enabling people to learn various health-related skills throughout life.

Sports clubs are places where young people, coaches and parents meet, and where all of these groups can develop various skills significant for health development. Kokko and colleagues [19] argue that the informal nature of the sports club as an educational setting is an advantage in this regard, and Engström [29] states that youth sport is one of our most important environments for young people’s socialization. Fraser-Thomas and colleagues [27] conclude that the role of sports organizations should be to design sports that develop better people rather than skilled individuals.

In many youth sports clubs, the coaches are volunteers; for instance, they can be parents with interest and engagement but often no formal education for the task. De Knop and colleagues [58] conclude that the lack of quality in guidance in sports clubs has to do with coaches and instructors having no qualifications for this specific task. Gould and Carson [65] add that youth sports coaches have little formal education, especially in the area of life skills, and one suggestion from De Knop and De Martelaer [49] is to reward sports clubs that work with qualified youth coaches. Another example to increase the likelihood of parents and parent-coaches creating a positive climate is the programme Minnesota PLAYS, which has been produced as a research-based curriculum for parents of young athletes. This programme will help parents to understand their roles in creating healthy, family-friendly youth sports [66].

Alongside the design of youth sports activities, the influence of parents and coaches is regarded as one of the most important factors affecting youth sports [27]. The coaches in the included studies are important authority figures for adolescents, although this influence can be conscious or unconscious, positive or negative [19]. A coach can influence the sports environment in other ways than just the design of the youth sports environment. Bloom [67] states that the role of youth sports coaches is complex and multidimensional. He enumerates 13 different roles: instructor, teacher, trainer, motivator, disciplinarian, substitute parent, social worker, friend, scientist, student, manager, administrator and publicity fundraiser. According to Fry and Gano-Overway [68], coaches can, by providing a caring climate, encourage more involvement in sports and get fewer athletes to drop out.

Rutten and colleagues [69] consider good relations with the coach and high levels of socio-moral reasoning to be a way to reduce antisocial behaviour. Guivernau and Duda [70] state that coaches need to be aware of their important role for young people’s moral functioning, but that parents and other people connected to the sport organization also can influence the moral climate. Guivernau and Duda [70] further report that when youth are faced with moral decisions in sports, it is the coach who influences them. Psychosocial coach training is associated with gains in self-esteem, especially for those who need it most [71]. Gould and colleagues [72] interviewed experienced and successful coaches and found that part of what made them successful was their having: well-defined coaching philosophies, with explicit life-skills development; the ability to form strong relationships with players; a variety of strategies for teaching life skills; an understanding of the environmental factors; and other individuals able to influence life skills developments that occur. When Fraser-Thomas and colleagues [73] compared comments about coaches by dropouts and by those still active, they found that it was important that coaches could communicate openly with their athletes.

Parents are also important in many ways in relation to developing skills, perhaps mostly by encouraging their children to participate in sport in a healthy way [73]. For example, parents, together with coaches, must play a more active role in deciding when a child is mature enough to understand when to specialize in a sport [55]. Positive parental interactions, support and encouragement are important, but too much pressure, such as in the form of criticism and high expectations, is not helpful [26,27]. A negative influence described earlier is angry parents [36].

In this review, the youth sports club as a health promoting setting for youth are studied, but the youth sports club could also be a health promoting setting for the coaches and the dedicated parents who have the chance to volunteer and acquire a new social network. Studies have shown, for example, that people who do volunteer work have better mental health [74].

#### Reorienting health services

In recent years the health sector’s level of responsibility for health promotion has increased beyond their earlier responsibilities. This suggests – in a health-promoting settings approach – that there is a need to support individuals and communities seeking a healthier way of living. It also opens channels between the health sector and other components, for example written prescriptions of physical activity or injury prevention in youth sports.

One area of the health-promoting settings approach that only to a lesser extent is connected to youth sports clubs in the review is health services. Sports injuries among young athletes are, however, a public health issue [75]. Timpka and colleagues [37] state that preventing injuries from occurring in the first place and thereby delivering safe sport environments should be a major goal for youth sports clubs, and should also be incorporated into public health promotion agendas. Since 2006, there has been an international non-profit programme for global promotion of sports safety: Safe Sports International (SSI). Their aim is to establish the sports injury problem on the global health policy agenda and to introduce sports safety as a component in the establishment of sustainable sports organizations [37]. In this matter, Frisch and colleagues [75] argue that educating coaches about sports injuries is an important issue, and that injury prevention should be a topic in coach training programmes. Weaver [76] further concludes that the cause of injuries is multifactorial and requires a holistically-structured prevention approach. This means that you have to look at injury prevention from different directions in terms of physical or social environment, primary, secondary, or tertiary prevention and changing the athlete’s behaviour and/or changing the environment [76].

### A framework for youth sports clubs as health-promoting settings

The second aim of this paper is to synthesize the results of the integrative review in terms of a framework for the youth sports club as a health-promoting setting. The review reveals that youth sports clubs have plentiful opportunities to be or become health-promoting settings. However, this is not something that happens automatically. To do so, the club needs to include an emphasis on certain important elements in its strategies and daily practices. The youth sports club needs to be a supportive and healthy environment with activities designed for and adapted to the specific age-group or phase. The adults, both coaches and parents, have to support the youth in a sound way, help them make good decisions, but not push them too hard. To help with these issues the club needs continuous deliberation on policies concerning visions and aims, as well as rights and duties for all its members. The contents of the policies further need to be closely related to the daily activities of the club. Finally, the review reveals that in order to become a health- promoting setting the youth sports club should not solely operate within its own setting. It needs to reach out to the surrounding community, including schools as well as local and national sports federations.

The starting point for our discussion of a framework for youth sports clubs as health promoting settings is, apart from the review, also the influence of previous work by Kokko and colleagues [19] and Dooris [14]. Kokko and colleagues [19] made an initial attempt to create a framework for the concept of health promoting sports clubs. Through a Delphi method, that is, a group consensus approach conducted with experts on health promotion and sports clubs in Finland, 22 standards for the health promoting sports club were created. Dooris’s [14] work further includes a multi-stakeholder approach for healthy schools, which elegantly emphasizes the diversity of motivations that may drive healthy settings work in a broad sense.

Taken together, Kokko and colleagues [19] and Dooris [14] reveal that a health-promoting setting is a complex network of internal and external actors; Figure 1 illustrates the result of our synthesis: a health-promoting youth sports club’s different internal and external actors as well as the settings with which the club could collaborate.

The internal actors in a health-promoting youth sports club are, of course, the young athletes themselves with their different types of coaches, parents, and siblings, and the club’s board (which often consists of engaged parents and coaches). However, to accomplish a transformation into a more healthy and supportive environment, it is important to focus on changing the whole organization [46] and becoming more professional [47]. This means that drafting a couple of policy documents about different areas is not enough, although it can be a good way to start working with the whole picture. Health-promoting policies in youth sports clubs can have explicitly health-promoting meanings like, for example, rules about alcohol and other drugs in sports clubs settings. However, there are also indirect effects of having policies, such as the support that volunteer leaders can feel when they have structured policies to lean on [77]. Policies can further affect both a sports club’s design and the parents’ and coaches’ attitudes. Figure 2 further illustrates the examples of the key issues from the review illustrated in what the different actors could work with to build a health promoting sports club.

A health-promoting youth sports club also needs to think about how its particular sport and training are designed. Are the practices within the club connected to youth’s socialization into sport [53]? Given that multisport participation has been shown to be advantageous for many reasons [25,29,54,58–61], can the members engage in more than one sport (or other activity) at the same time?

The efforts to change the youth sports club to a healthier and more supportive environment will probably also cause the daily activities of the club to change. The active work must, of course, take place within the club, with all the internal actors represented, to secure broad democratic support for the changes [48], though it is also important not to reinvent the wheel. Other sports clubs, both in the neighbourhood but practising other sports, and also non-local clubs practising the same sport, may have started a similar process. Learning from and helping each other is an interesting and promising way to work. This could be compared to the concept of “communities of practice”, which involves the idea that “groups of people who share a concern or a passion for something they do, learn how to do it better as they interact regularly” [cf. 78, 79, p. 1]. There are also other external actors that can help, for example national sport governing bodies, sports organisations, and educational associations.

In countries like Sweden, both district sport federations and the Swedish sports education organization are of great importance when it comes to educating coaches, parents and youth, but also when developing policies in the clubs. The municipality or region where the sports club is located is also an important external actor. On the other hand, some municipalities in Sweden are discussing making it a condition for receiving financial support that youth sports clubs, for example, have an alcohol policy; and the number of aims attached to the support to sports clubs, such as equality, integration and drug prevention, has been growing in Sweden [80]. A risk with these kinds of conditions and requirements for financial support is that this kind of top-down perspective can force youth sports clubs to have policies that are only products of the drawing board. Sponsors could also be regarded as actors external to youth sports clubs. However, many sports clubs are discussing whether it is inappropriate to accept sponsorship from some kinds of companies, with alcohol companies especially being discussed.

A single youth sports club, taken in isolation, can appear to be a quite small health-promoting setting. However, taken together, sports clubs and diverse forms of organized youth sports reach a quite substantial portion of the young population, which makes the youth sports club a setting with great potential. Nevertheless, it is important that the clubs cooperate with other settings, such as schools, communities and the health sector. One example is that the youth sports club could be a new arena for parent interventions, although studies have shown that it can be quite difficult to get parents to participate in a parent intervention at school [81]. The Swedish project “ÖPP (Örebro Prevention Program) in club activities” is an interesting example run by a district sports federation in a municipality in Sweden. Still in its early stages, this project will be testing a manual-based parent intervention which has previously been tried in schools in order to, among other things, maintain restrictive attitudes toward youth alcohol use [82].

Comparing Kokko and colleagues’ [19] standards for health-promoting sports clubs with the results of this review and synthesis discloses many similarities and also some differences. Also, Kokko and colleagues [19] view health-promoting sports clubs more broadly in terms of the use of sports clubs’ activities for promoting the health for youth. They also argue that a conceptualization of the health-promoting sports club is a combination of previous theory and information on the daily activities of sports clubs, something we hope to contribute to with this review. Their standards are as our result based on the strategic headings of the Ottawa Charter, with rather similar content, although with some differences in what goes under which category. Our framework further emphasizes the plethora of actors in the health-promoting youth sports clubs’ network in relation to the key issues for a health-promoting youth sports club.

Shortcomings of our review we have noticed are that the keywords used in the database search could have been extended to include also child/children’s sports in addition to youth sports. In some cases this could have lead to more studies being included. Further, with other types of keywords, such as for example healthy psychosocial development and positive youth development, more sports psychology studies could have been found. This can be seen as a potential for further reviews in this setting. Although the articles were peer-reviewed and published in international journals, there may be a need for quality assessment of the publications. However, this integrative review did aim at uncover new ways of conceptualizing the research field and not to aggregate data to produce statistical cause- and effect relationships.

## Conclusion

Sport is, of course, not a monolithic entity [83]. Different sports have different requirements and different traditions, which makes all sports settings in some way unique; and with regard to health, health promotion can be seen as just another demand on already over-burdened coaches and board members. We would argue that health can scarcely be separated from the other goals of a club, but it is not, of course, always seen as the primary goal of youth sports clubs. Instead we agree with Fraser-Thomas and Côte [26] that if researchers and practitioners work together in a collaborative manner, it is more likely that young people will experience numerous positive outcomes through participation in sport, not least in relation to health.

However, the review and the previous synthesis have arrived at the conclusion that to become a health-promoting setting, a youth sports club needs to take a comprehensive approach to its activities, aims, and purposes. It is also important that the young athletes and their activities are put at the centre of who the club is seen as being for. In this way, the club can identify and cultivate the supportive environment necessary for it to be or become a health promoting setting in terms of what we argue to be *youth sports for health*.
